# Space-time variations in child mortality in a rural South African population with high HIV prevalence (2000–2014)

**DOI:** 10.1371/journal.pone.0182478

**Published:** 2017-08-24

**Authors:** Boikhutso Tlou, Benn Sartorius, Frank Tanser

**Affiliations:** 1 Discipline of Public Health Medicine, School of Nursing and Public Health, University of KwaZulu-Natal, Durban, South Africa; 2 Africa Centre for Health and Population Studies, University of KwaZulu-Natal, Mtubatuba, South Africa; 3 Centre for the AIDS Programme of Research in South Africa–CAPRISA, University of KwaZulu-Natal, Durban, South Africa; University of Tennessee Knoxville, UNITED STATES

## Abstract

**Objective:**

The aim of the study was to identify the key determinants of child mortality ‘hot-spots’ in space and time.

**Methods:**

Comprehensive population-based mortality data collected between 2000 and 2014 by the Africa Centre Demographic Information System located in the UMkhanyakude District of KwaZulu-Natal Province, South Africa, was analysed. We assigned all mortality events and person-time of observation for children <5 years of age to an exact homestead of residence (mapped to <2m accuracy as part of the DSA platform). Using these exact locations, both the Kulldorff and Tango spatial scan statistics for regular and irregular shaped cluster detection were used to identify clusters of childhood mortality events in both space and time.

**Findings:**

Of the 49 986 children aged < 5 years who resided in the study area between 2000 and 2014, 2010 (4.0%) died. Childhood mortality decreased by 80% over the period from >20 per 1000 person-years in 2001–2003 to 4 per 1000 person-years in 2014. The two scanning spatial techniques identified two high-risk clusters for child mortality along the eastern border of the study site near the national highway, with a relative risk of 2.10 and 1.91 respectively.

**Conclusions:**

The high-risk communities detected in this work, and the differential risk factor profile of these communities, can assist public health professionals to identify similar populations in other parts of rural South Africa. Identifying child mortality hot-spots will potentially guide policy interventions in rural, resource-limited settings.

## Introduction

Childhood mortality is a major public health problem in many developing countries. Half of all deaths of children under age 5 occur in only six developing countries (India, Nigeria, China, Pakistan, Democratic Republic of Congo, and Ethiopia), with high childhood mortality rates being concentrated primarily in sub-Saharan Africa (SSA) [1]. Within this region, childhood mortality in South Africa decreased from 60 deaths per 1000 live births in 1990 to 41 deaths per 1000 live births in 2015, with an annual rate of reduction of 1.6. A study conducted in the country’s rural KwaZulu-Natal (KZN) Province showed that between 1990 and 2000, infant mortality increased from 43 to 65 per 1000 live births, and under-five mortality from 65 to 116 per 1000 live births [2]. The Millennium Development Goal(MDG)4 target for 2015 was 20 deaths per 1000 live births [3], was not being met despite the large reduction in child mortality from 1990 to 2015. In order to achieve the global MDG 4 target, the under-five mortality rate would have had to decrease by 15.6% for 2012–2015, much faster than the 3.9% achieved over 2005–2012 [4]. This lack of progress could be due to HIV remaining the largest cause of fatalities in children younger than five, accounting for 40% of deaths globally [3]. However, the rate of improvement in HIV care, in terms of access to antiretroviral treatment (ART) in South Africa since 2005, has been impressive, being the fourth-fastest globally, and second only to Rwanda among African countries [5].

Understanding the impact of place, person and time on health is a key element of epidemiologic investigation, with spatial methods being useful to analyse geo-referenced health-related data [6]. In exploring the distribution of childhood mortality in South Africa, most studies have often focused on provincial level differentials [7] ignoring the impact of time. Even though studies on provincial level differentials are very important and have highlighted the magnitude of childhood mortality, they tend to ignore or mask considerable disparities in health that may exist within the population at different levels of geography. Space time clustering and the risk profile of childhood mortality has not previously been done at a truly local level in rural KZN.

The identification and characterisation of geographical clusters of high mortality is an important public health policy issue that has received limited attention, especially in rural South Africa. The spatial distribution of child mortality is not uniform across countries, localities or population groups. Wide disparities exist among and within groups even in the same country [8], and South Africa is no exception in this regard. The country is committed to reducing child and maternal mortality, this being reflected in the Negotiated Service Delivery Agreement that was signed in 2010, and which identifies their reductions (as well as in the prevalence of Tuberculosis(TB) and HIV) as key strategic priorities for the health sector [9]. Spatial disparities in the rates of childhood mortality vary by province, magisterial district, district municipal council and place of residence (urban-rural) [10]. Mapping these disparities will make targeting high risk areas a feasible way of reaching the high risk populations.

The aim of this study was to identify space-time child mortality clusters at a micro-geographical level in this typical rural population, and to describe the associated risk profile of the clusters. Here we present the results from a population based in rural KZN, in which approximately 50 000 study participants were geo-located to an accuracy of <2 m. Spatial techniques were applied to investigate the micro-geographical variation in childhood mortality clustering at a truly local level. The intention is to provide guidance regarding the distribution of health services and other spatially-targeted interventions for childhood mortality reduction and resource allocation in rural South Africa that may be applied to the broader SSA context.

## Methods

Ethical approval was received from the Biomedical Research Ethics Committee (BREC) of the University of KwaZulu-Natal (BE 169/15).

### Study area

**Fig 1:**
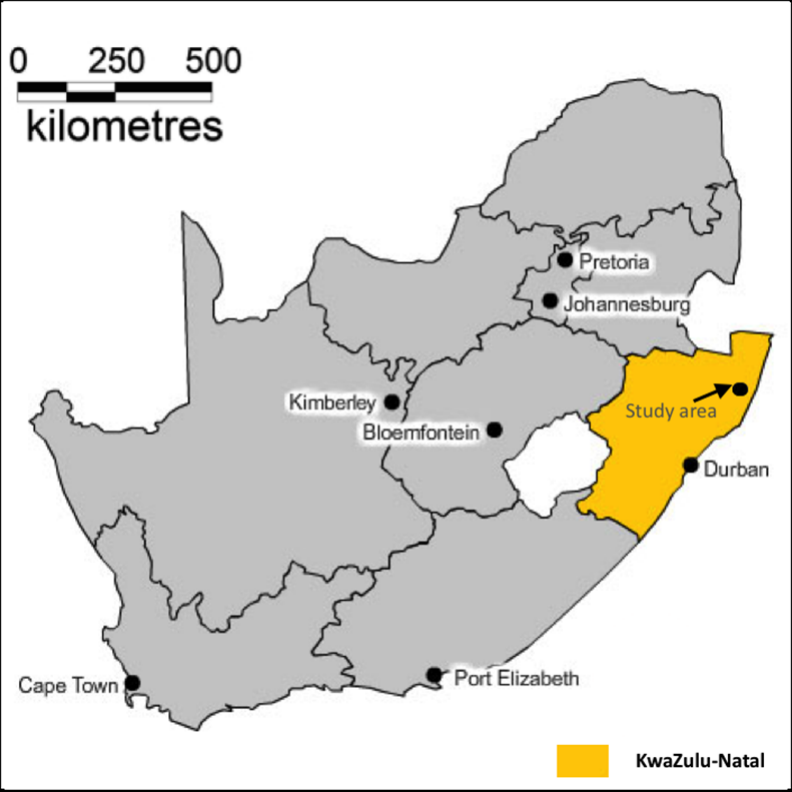
Location of the Africa Centre’s study area in KwaZulu-Natal Province, South Africa.

The study area (Fig 1) is located near the market town of Mtubatuba in the UMkhanyakude District of KwaZulu-Natal Province, South Africa [11]. The demographic surveillance site (DSS) was founded in 2000 and is 438 km^2^ in size [12]. The area has an approximate population of 87 000 Zulu people who are members of around 12 000 scattered households, and is characterised by distinct variations in population densities (20–3000 people/ km^2^). Whilst predominantly rural, the area contains an urban township and informal peri-urban settlements, as is typical in many rural areas of the country [11]. Adult unemployment rates are high, and the district has one of the lowest scores in KwaZulu-Natal with regard to the United Nation’s Human Development Index [12]. The principal sources of income for most households is waged employment, and state pensions and grants [11].

### Study population

We conducted a longitudinal analysis on children (<5 years of age) registered in the Africa Centre surveillance area who were born, residing or in-migrated as residents in the Africa Centre study area between 2000 and 2014. All deaths of children under the age of five years that occurred between 2000 and 2014 were included in the analysis, and is described in detail elsewhere [11], [13], [14], [15], [16].

### Detection of spatial clusters

Child mortality cluster detection was initially performed using purely spatial and spatial–temporal statistics separately. Detection of under-five spatial mortality clusters was done using the Kulldorff spatial scan statistic implemented in SaTScan [17] using the Poisson distribution, and p values were generated across 999 Monte Carlo replications (alpha <0.05). The circular spatial window scans the studied region with a large number of circles, and detects the most likely, signifi-cant cluster(s) represented by these circle(s). Circular windows are best for detecting small, compact clusters [17] We tested various permutations (10% -50%) for the maximum spatial cluster size. The most robust was 25% while 50% was too homogeneous.

A likelihood ratio test is used to determine a p-value for the possible clusters in order to reject the null hypothesis (no clustering). However, one limitation of the Kulldorff scan statistic is that it uses a circular window to define the potential cluster areas and thus cannot detect irregularly shaped clusters, with the geographical distribution of health outcomes generally being non-circular. Thus, the Flexible spatial scan statistic (Tango spatial scan statistic implemented in FleXScan [18]) was used for the analysis, in which the detected cluster is allowed to be flexible in shape, while at the same time, it is confined within relatively small neighbourhoods of each region. We applied the Poisson model using the (Log likelihood ratio) LLR with Restriction, with a preset parameter for restriction ‘Alpha’. We used the default value of 0.2. This statistic avoids detecting undesirably large clusters, and improves calculation time. In FleXScan, this parameter setting relates to the maximum cluster size (*K* is a pre-specified maximum length of cluster). The Current implementation of FleXScan does not suggest a value greater than 30, [18] which is what we used. We also employed 9999 Monte Carlo replications. However, the method is not practically feasible for larger cluster sizes, as it only works well for small to moderate cluster of up to 30 homesteads [17]. The data was aggregated into a grid of size 0.01 degrees by 0.01 degrees, which resulted in 705 grid cell aggregations for FleXScan, given its current limitations of being able to search a maximum of 30 adjacent nodes, which was assumed in this analysis. Lastly, we did a space- time analysis using both the Kulldorff and Flexible spatial scan statistics. We did an analysis from 2000–2014 but there was no evidence of spatio-temporal clustering. Then, we assessed spatio temporal clustering from 2000–2004 a pre rollout of antiretroviral therapy period. We tested various permutations for determining the maximum spatial cluster size (10% -50%).The most robust was 25% while 50% was too homogeneous.

### Mortality rate calculation and logistic regression

The data analysis was conducted using STATA software (version 14). The mortality rate estimation was calculated using the number of deaths and person-years lived by the children for each year, and the Confidence intervals (CI) for mortality rates were computed using the exact CI based on the Poisson distribution. The multivariable logistic regression was used to compare the characteristics (risk factors) of clusters versus non-clusters, adjusting for clustering using Huber’s adjustment, which ensures that standard errors are consistent even if the residuals are heteroscedastic. The traditional standard error estimates based on maximum likelihood from independent observations are not appropriate for data sets with cluster structure, since observations in the same clusters tend to have similar characteristics and are more likely correlated with each other. Huber robust standard error estimates are very important and useful for data sets with cluster structure and take intra-cluster correlation into account during analysis. We used the Hosmer & Lemeshow test and Receiver Operating Characteristic(ROC) to assess the goodness fit of the logistic model.The unit of analysis for the logistic model were individuals.

## Results

### Study population and mortality

**Fig 2:**
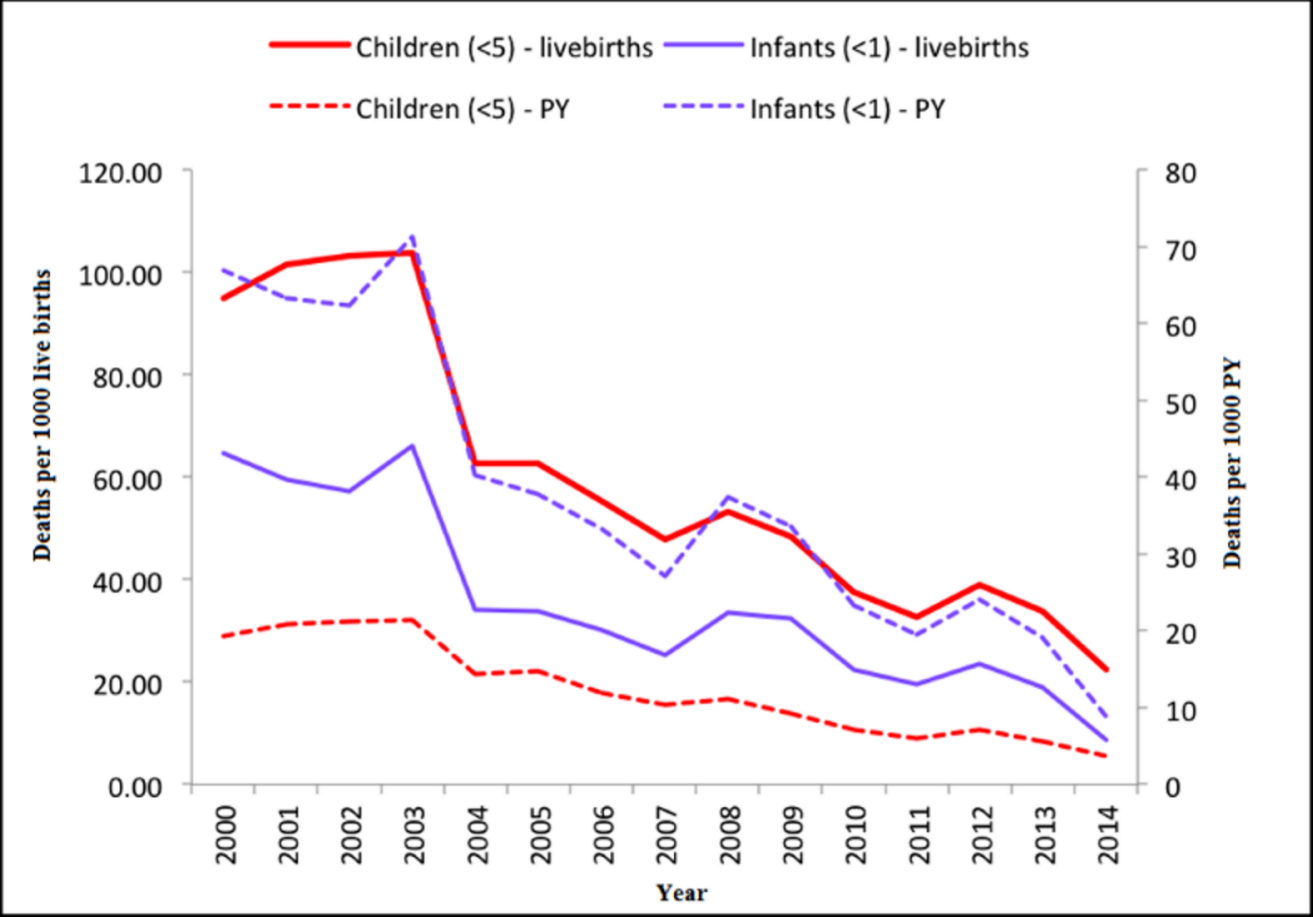
Infant and child mortality rates per 1000 live births and person-years between 2000 and 2014.

The study population comprised 49 986 children, with an approximately equal gender ratio (25 241 males [50.5%] to 24 743 females [49.5%], 2 unknown). Overall, there were 2 010 (4.0%) deaths from a total of 49,986 children between 2000 and 2014. The overall 15 year period child mortality rate (in person-years) was 13.1 deaths per 1000 child person-years, while the overall child mortality rate (scaled by live birth) was 61.6 deaths per 1000 live births. The trends for infant and child mortality by year are shown in Fig 2, which indicates that both mortality rates decreased significantly over the study period. The five leading causes of child deaths in the Africa Centre surveillance area were: acute respiratory infection, including pneumonia (39.5%), HIV/AIDS related (25.91%), neonatal pneumonia (3.52%) diarrhoeal diseases (3.23%) and pulmonary tuberculosis (2.68%).

### Spatial clusters of child mortality

**Fig 3:**
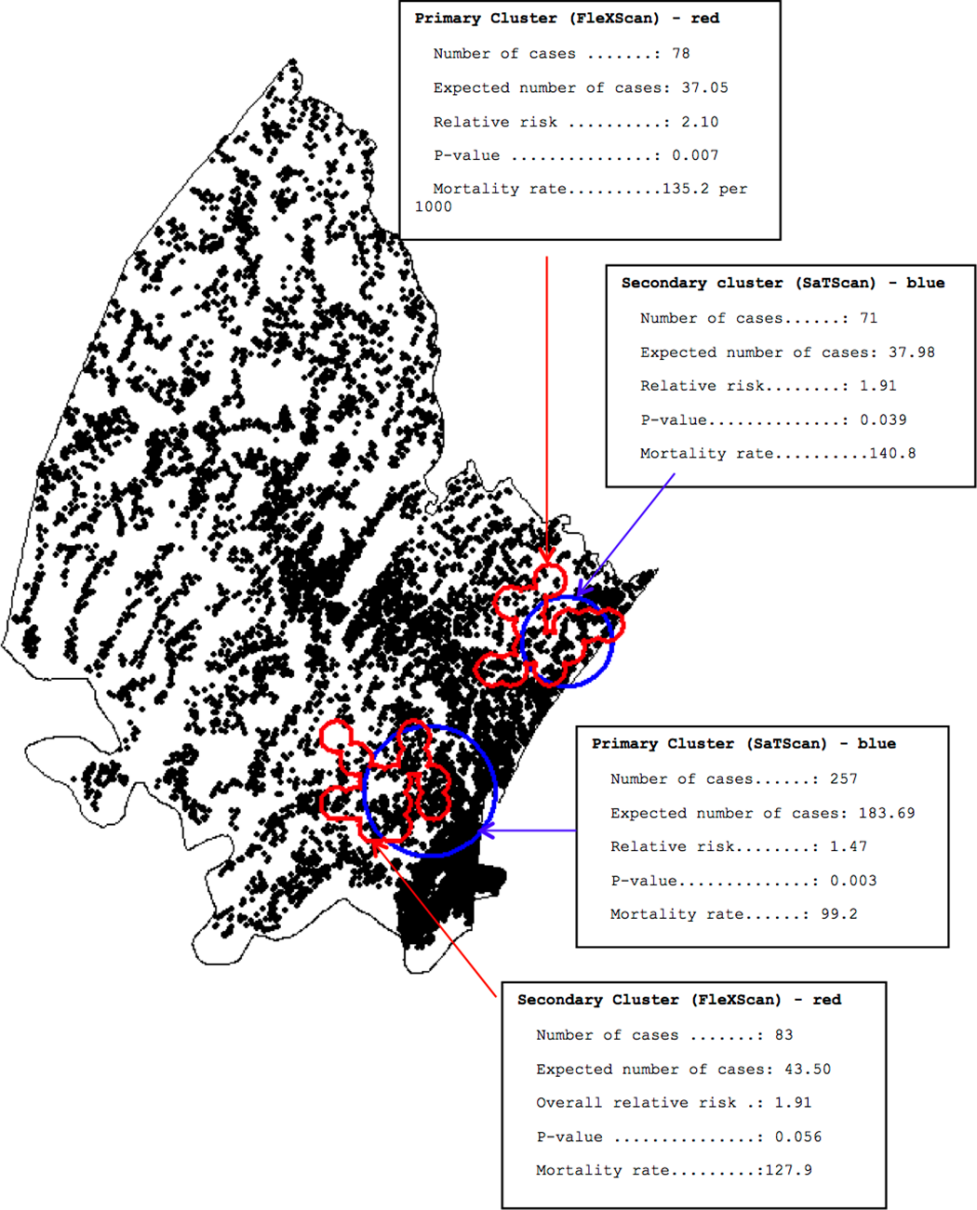
Spatial clustering of childhood mortality (Kulldorff and Tango spatial scan statistics) [maximum 25% population at risk in scan window].

**Fig 4:**
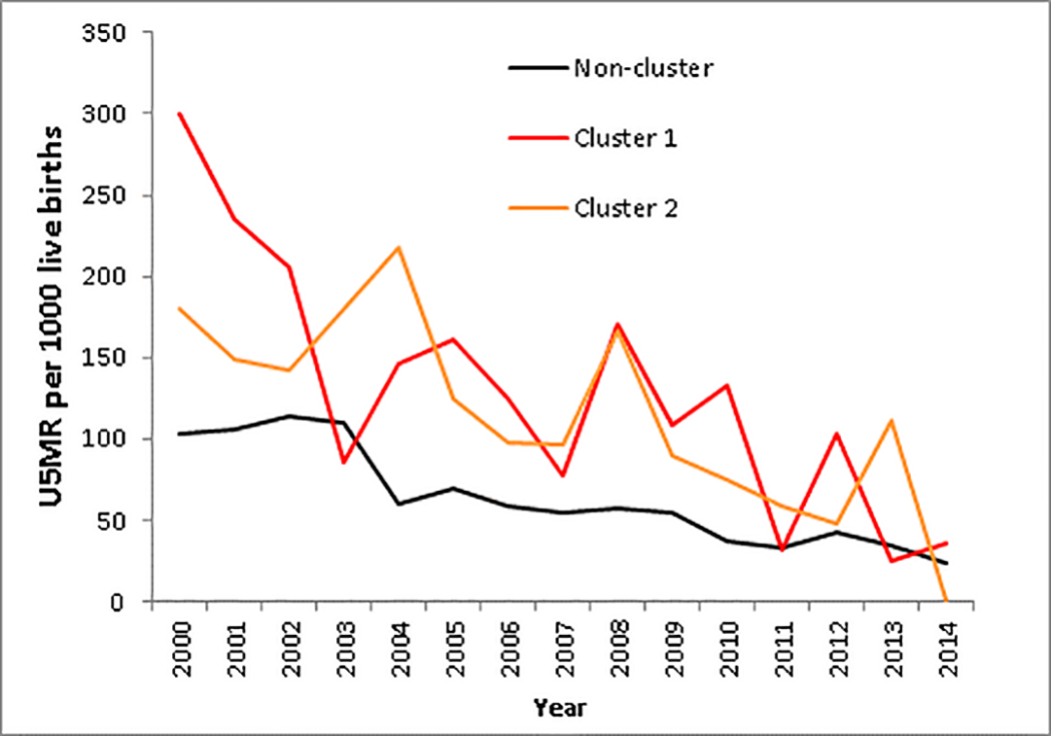
Child mortality rates per 1000 live births per year by cluster and non-cluster.

A significant high-risk primary cluster using the circular Kulldorff spatial scan statistic, was identified along the south-east boundary of the site near the urban centre of the study area, with a relative risk of 1.47 (99.2 deaths per 1000 live births). A statistically significant secondary hotspot was located further north along the eastern boundary, with a relative risk of 1.91 (140.8 deaths per 1000 live births) (Fig 3). The flexibly shaped spatial scan statistic also detected two significant high risk clusters in similar locations to the Kulldorff clusters, but with more refined extents (Fig 3). The relative risks for the primary and secondary clusters were 2.1 (135.2 deaths per 1000 live births) and 1.9 (127.9 deaths per 1000 live births) respectively. Trends in child mortality from 2000–2014 in the clusters versus the non-clusters followed the similar declining trend of the study period, albeit at a high relative level in the clusters identified earlier in the study period, with approximate convergence around 2014 (Fig 4). The mortality trends in Fig 4 were assessed from the clusters identified, and child mortality was observed to decrease over time from year 2000 to 2014.

### Spatio-temporal clustering of child mortality

**Fig 5:**
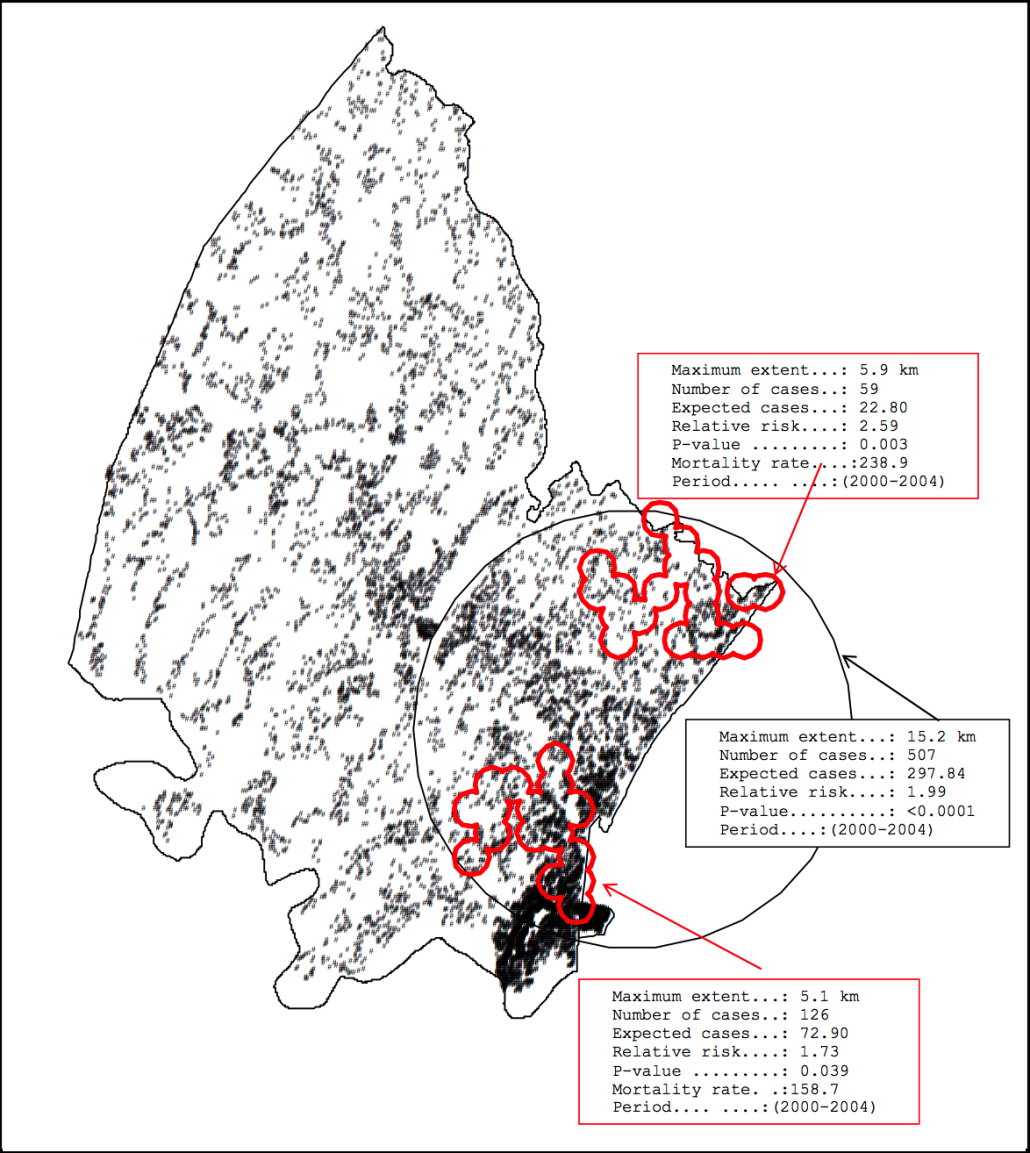
Space-time clustering of child mortality in Africa Centre study area, 2000–2004.

In addition, we used the Kulldorff space-time scan statistic to identify clusters of child mortality in space-time (Fig 5). Significant space-time clusters (15.2 km in extent) were identified from 2000 to 2004 along the eastern border of the study site, with a relative risk of 1.99, and contained 507 deaths (expected deaths = 298) with a p-value of 0.003.Also we used the flexible spatial scan static to identify space time child mortality clusters. We found two significant space time clusters in the south eastern (5.9 km in extent) and south eastern (5.1 km in extent) part of the study site. The south eastern cluster had a relative risk of 2.39, and contained 59 deaths (expected deaths = 23) with a p-value of 0.003, whilst the southern cluster had a relative risk of 1.73 and contained 126 deaths (expected deaths = 73) with a p-value of 0.039. The application of the Tango cluster statistic (FlexScan) for the period 2000–2004 suggested that approximately a third of these excess deaths may have been in the lower region of the larger cluster.

### Characterising the communities with high child mortality

We assessed the risk factors for both the Kulldorf and Tango clusters using the multivariable logistic regression. The results were similar in both classification clusters, but we presented the FleXScan results from the spatial clustering in Table 1 since the Kulldorf spatial scan is circular and there is a high possibility of the inclusion of non-risk areas in the cluster. Lastly, we compared the risk factor profile of the clusters versus the non-cluster to identify potential underlying reasons for the increased mortality risk in these hotspots using a logistic regression (Table 1). The covariates were divided into direct and underlying causes at the household/community and district levels, factors that have been shown to be associated with child mortality in other settings. We assessed for collinearity between predictors using the correlation matrix and there was no prominent collinearity between the predictors as shown in S1 Table. We firstly did a univariable analysis to explore the unadjusted association between each covariate and the response variable (presence of a cluster). A p-value of smaller than 0.05 and variables of known clinical relevance were included for further multivariable analysis. Only two direct causes (deaths due AIDS/TB and deaths due to communicable diseases) and one underlying cause at household/community and district level (source of drinking water) were put into the final model after assessing multicollinearity and variables were uncorrelated with each other. We used the stepwise automatic selection procedure to select the predictors for our final model. The final model indicated deaths due to HIV/TB and source of drinking as significant covariates. We also assessed interaction effects between the significant covariates and none were significant. The log likelihood chi-square indicated that the model with a better fit than all the others was the one presented in the multivariable section of Table 1. The Hosmer & Lemeshow test of the goodness of fit also suggested the model was a good fit to the data as p = 0.813 (>0.05).However, the model did not have a great predictive ability as the area under the ROC was 0.5586.

**Table 1:** Logistic regression results showing profiles of children/household in clusters vs. non-cluster.

		Univariate			Multivariable		
Characteristic		Odds ratio	95%CI	p-value	Odds ratio	95%CI	p-value
**Direct Causes**							
**Deaths due to AIDS/TB**	Yes	1.98	1.40–2.81	<0.001	7.54	7.49–7.59	<0.001
	No	1			1		
**Deaths due to unknown causes**	Yes	1.24	0.45–3.42	0.680			
	No	1					
**Deaths due to non-communicable diseases**	Yes	1.01	0.13–7.55	0.993			
	No	1					
**Underlying causes at household/community and district levels**							
**Source of drinking water**	Borehole	1.18	0.71–1.95	0.517	1.06	0.99–1.12	0.054
	Well	1.28	0.75–2.17	0.365	1.18	1.16–1.21	<0.001
	Surface(lakes and dams)	1.71	1.23–2.36	0.001	1.70	1.70–1.72	<0.001
	Other	1.31	0.91–1.89	0.142	1.38	1.37–1.39	<0.001
	Piped water	1			1		
**Mother HIV Positive**	Yes	1.08	0.81–1.45	0.584			
	No	1					
**Father HIV Positive**	Yes	1.24	0.62–2.49	0.538			
	No	1					
**Wealth Index**	Poor	0.95	0.71–1.29	0.787			
	Middle	0.90	0.64–1.26	0.547			
	Rich	1					
**Ever vaccination**	No	0.99	0.81–1.22	0.968			
	Yes	1					
**Mother education**	None	0.57	0.21–1.55	0.272			
	Primary	0.76	0.55–1.04	0.084			
	Secondary or more	1					
**Birth order**	First	0.99	0.82–1.20	0.964			
	Five or higher	0.91	0.64–1.29	0.596			
	Second, Third and Fourth	1					
**Distance from nearest clinic in km**	>10	1.27	0.55–2.93	0.568			
	<10	1					

Clusters had significantly higher adult HIV Prevalence, deaths due to HIV/AIDS and /or TB, and deaths due to communicable causes as compared to the non-cluster. In addition to that children whose source of drinking water was from surface bodies (lakes and dams) were more likely to be in the clusters as compared to the non-clusters. The primary cluster based on the flexible scan statistic had a significantly higher adult prevalence of HIV among those >15 years of age (p<0.001): 26.8% in cluster 1, 22.3% in cluster 2 and 21.6% in the remainder of the site (non-cluster). There was no evidence of significant differences between the clusters and non-cluster with regards to mother or father’s HIV status, (although higher in the clusters), wealth index, birth order parents education, distance to the nearest clinic and vaccination coverage.

## Discussion

The results of the study show a considerable decline in child mortality in the Africa Centre Demographic Surveillance Area (DSA) from 2000 to 2014. This is consistent with other findings in India and Nigeria [19], KwaZulu-Natal (ACDIS [20, 21]; Amajuba district [22]) and South Africa [23], and reflects the roll-out of ART and improvements in HIV services. Significant child mortality hot-spots were identified on the eastern boundary of the site in peri-urban communities near the main highway in the high HIV-prevalence and incidence communities [24]. Previous studies have demonstrated a strong ecological relationship between proximity to roads and HIV prevalence among women attending antenatal clinics in this setting [12]. These findings are supported by ecological observations made in this area and other parts of Africa and suggest that individuals living in communities with better access to transport and transport routes are at higher risk of infection [14]. Similarly, a study done in Rakai District(Uganda) indicated an association between distance to main roads and increase in both HIV incidence and genetic complexity of the virus, [25] thus households located near a main road were twice as likely to be infected with HIV. The possible reason suggested was that communities along main roads in Rakai District, would provide an environment for increased social and sexual interaction. Also, the association of higher HIV-1 prevalence with proximity to paved roads was demonstrated, suggesting that HIV-1 transmission tends to follow lines of transportation, communication, and social interaction.Space–time analysis identified one significant cluster of higher mortality rates from 2000 to 2004 located in the eastern border of the study site.

Our work represents the first time that a micro-geographical spatio—temporal analysis has been performed on child mortality outcomes in an African population. Another strength of this study is that there was longitudinal follow-up of a complete population over the period in an area where the public-sector ART programme has been scaled up. However, as a limitation, we tested various permutations for determining the maximum spatial cluster size and this introduces the problem of multiple testing. Another limitation of the study is that it is not always easy to collect data in rural parts of developing countries, making it difficult to achieve a record of all deaths in this age-group. Census update rounds are conducted once a year. This makes an undercount of abortions, stillbirths and early infant deaths likely. The implication is whether underreporting of cases, or the incomplete recording of events (deaths), could have had an impact on our results.To minimize perinatal deaths missed, the last child born to each woman is printed on the populated census form, and fieldworkers use this prompt to probe for any pregnancies since this last birth. In addition, starting 2006, fieldworkers ask if any woman in the household is currently pregnant, and if so the expected delivery date [26]. Although we cannot rule out that some deaths remained unreported, we do not have evidence of differential underreporting of cases between villages. The non-existence of clusters with significantly lower total childhood mortality in the study area indicates that there was no systematic underreporting in some villages.

Also, some members of the population have changed their place of residence within the observation period due to migration. Movement from one household to another within the study site is common. However, tracking of internal migrants to link the household of origin and destination for each move is made, thereby ensuring a single unique identifier for each individual, and removing the potential of one person being concurrently registered in two households [27].Also, if a subject is absent, the field workers make up to four repeat visits to the same household. If a subject no longer lives in the household, the field worker hands the case to a specially trained tracking team that attempts to find the individual in his or her new residence which may be as far as Johannesburg or Durban [28].

**Fig 6:**
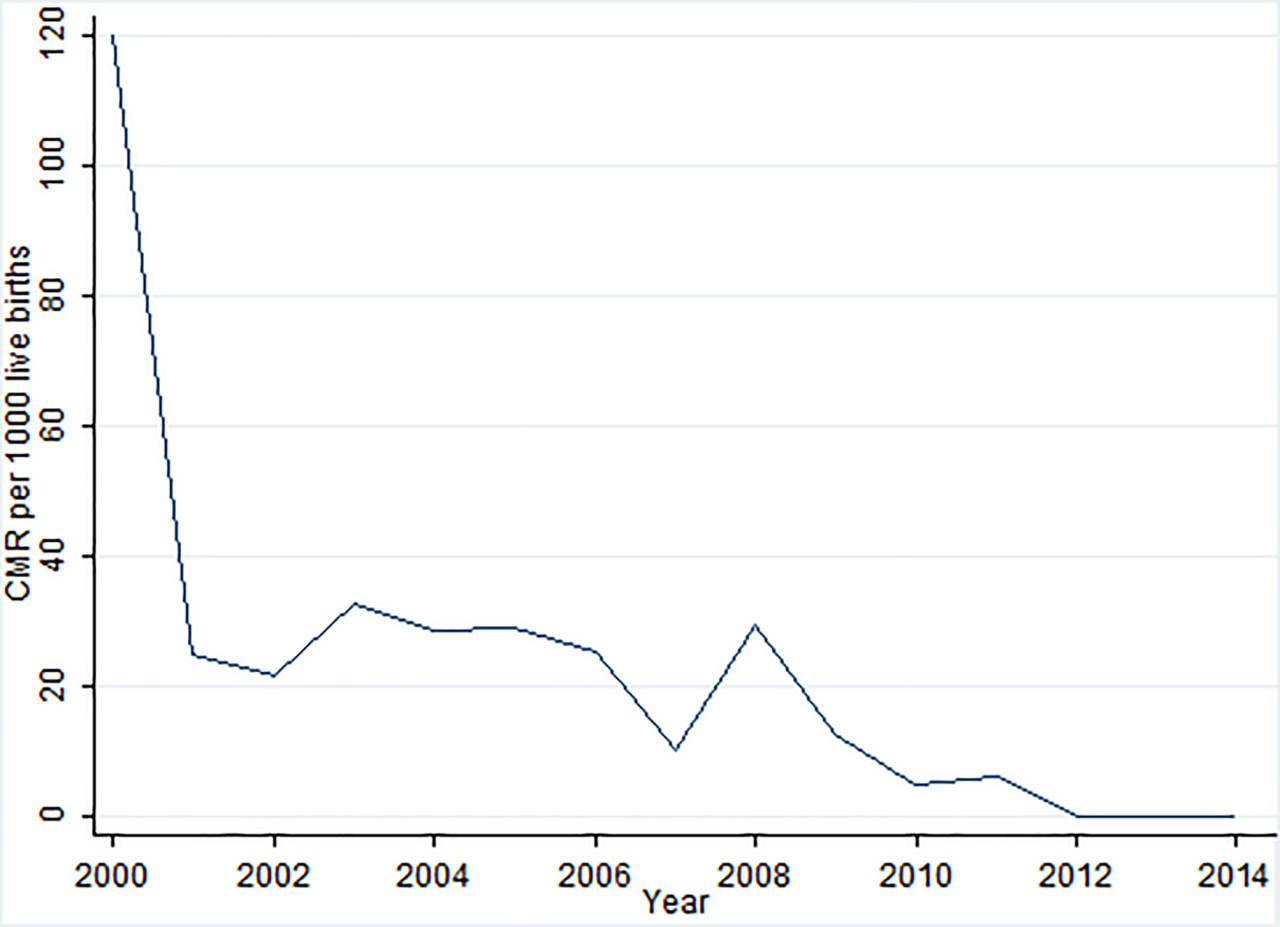
Changes over time on child mortality rates per 1000 live births caused by HIV/AIDS during the study period.

Globally, the number of deaths of children under 5 years of age fell from 12.7 million in 1990 to 6.3 million in 2013 [29]. The large decline in childhood mortality observed in this population most likely reflects the combined impact of prevention of mother-to-child HIV transmission and the scale-up antiretroviral therapy provision (started in 2004 [30]) in the district. Fig 6 provides the empirical evidence of changes over time on child mortality rates per 1000 live births caused by HIV/AIDS during the study period. Our findings are also in line with those from other recent regional and local studies [31, 32]. These trends suggest progress towards MDG4, although further progress will be needed to fully attain this goal. Identifying mortality hotspots clearly demonstrates significant spatial and temporal heterogeneity of child mortality, even in a small and relatively homogenous geographic area. This finding is similar to what other studies in the Sub-Saharan region [33] and Africa Centre study area [34, 35] have found.

Attaining the now outdated MDG 4 and achieving the new sustainable development goals will require more rapid scale-up of key effective and affordable interventions, such as: care for new-borns and their mothers; infant and young child feeding; vaccines; prevention and case management of pneumonia, diarrhoea and sepsis; malaria control; and prevention and care of HIV/AIDS patients [36]. Furthermore, it is important to note that child mortality is heterogeneous in this area, and targeting interventions to these hotspots could therefore have a substantial impact. Thus, the results suggest that a one-size-fits-all intervention strategy may not be effective in such a situation where marked variations in socio-geographical context exist [37]. However, we should not be misled into thinking that a decline in child mortality is only due to a decline in HIV prevalence, as there may be other compounding factors. As a means of combating child mortality, policy makers should consider increasing the number of mobile clinics in this and similar communities in rural Africa.

The identification of child mortality clusters in this rural set-up will potentially guide policy interventions in rural, resource-limited settings. Past studies have indicated that women who lose a child have a higher chance of subsequent child losses, intervention programmes to lower infant mortality should not only focus on individuals but on their children, families and communities as well [38]. The link between HIV prevalence and death clustering suggests that reducing mother-to-child transmission may lower death clustering and infant mortality levels. The government should focus more on key preventive measures on improving water, sanitation and hygiene, which would contribute toward reducing diarrhoea related child deaths in the post-MDG era. Also, comprehensive policies for prevention, care, and treatment of HIV infection and TB are essential to reduce HIV/AIDS and TB related deaths. Pneumonia can be prevented by immunization, adequate nutrition, and by addressing environmental factors in under 5 children.

## Conclusions

This study has revealed the existence of clear spatial and spatial–temporal clusters of childhood mortality in this typical rural South Africa, and sub-Saharan African population. The existence of mortality clusters indicates the presence of variability in health risks, and has implications that should be addressed for better overall population health. Identifying mortality disparities by geographic area provides evidence for the need for spatial health planning, and can assist health policymakers to focus on high-risk areas for prevention programs and health care delivery, thereby effectively allocating public health resources for better health outcomes. This study may be regarded as a first step in prioritizing areas for analytical studies.

## Supporting information

S1 Table This is the correlation matrix between predictor variables used in the model. (DOCX)

S1 Appendix (DOCX)
