# Supplementary material for: Space-time variations in child mortality in a rural South African population with high HIV prevalence (2000–2014)
Source: PLoS One. 2017 Aug 24;12(8):e0182478. doi: 10.1371/journal.pone.0182478 (PMC5570377; doi:10.1371/journal.pone.0182478)
Supplement: S1 Table — (DOCX) [file pone.0182478.s001.docx]

**S1 Table : Correlation Matrix**

| **Variable** | **Correlation matrix of coefficients** | | | | | | | | | | **Diagnostics** | |
| --- | --- | --- | --- | --- | --- | --- | --- | --- | --- | --- | --- | --- |
|  | Source of drinking water | Death due to AIDS/TB | Death due to unknown diseases | Death due to non-communicable diseases | Mother HIV Positive | Father Hiv Positive | Wealth index | Mother education | Distance to the nearest clinic | Birth order | VIF | Tolerance |
| Source of drinking water | 1.00 |  |  |  |  |  |  |  |  |  | 1.02 | 0.98 |
| Death due to AIDS /TB | -0.03 | 1.00 |  |  |  |  |  |  |  |  | 1.02 | 0.98 |
| Death due to unknown causes | -0.06 | 0.01 | 1.00 |  |  |  |  |  |  |  | 1.01 | 0.99 |
| Death due to non-communicable diseases | 0.03 | 0.013 | 0.002 | 1.00 |  |  |  |  |  |  | 1.03 | 0.97 |
| Mother HIV Positive | -0.05 | 0.06 | 0.021 | -0.03 | 1.00 |  |  |  |  |  | 1.21 | 0.83 |
| Father HIV Positive | -0.02 | 0.10 | 0.008 | -0.07 | -0.40 | 1.00 |  |  |  |  | 1.22 | 0.81 |
| Wealth index | -0.07 | -0.024 | 0.04 | -0.03 | 0.011 | -0.03 | 1.00 |  |  |  | 1.10 | 0.91 |
| Mother education | 0.02 | 0.050 | 0.025 | 0.048 | -0.04 | -0.01 | -0.24 | 1.00 |  |  | 1.03 | 0.97 |
| Distance to the nearest clinic | 0.02 | 0.031 | 0.034 | 0.035 | 0.002 | 0.08 | -0.11 | 0.023 | 1.00 |  | 1.14 | 0.88 |
| Birth order | 0.0063 | -0.07 | -0.028 | -0.03 | 0.015 | 0.08 | -0.064 | -0.22 | -0.52 | 1.00 | 1.09 | 0.91 |
