# Supplementary material for: Space-time variations in child mortality in a rural South African population with high HIV prevalence (2000–2014)
Source: PLoS One. 2017 Aug 24;12(8):e0182478. doi: 10.1371/journal.pone.0182478 (PMC5570377; doi:10.1371/journal.pone.0182478)

**S1 Appendix**

**Table A: FLEXSCAN MULTIVARIABLE LOGISTIC REGRESSION**

|  |  | **Univariate** | | | **Maximum likelihood** | | | | **Huber’s Adjustment** | | | |
| --- | --- | --- | --- | --- | --- | --- | --- | --- | --- | --- | --- | --- |
| Characteristic |  | Odds ratio | 95%CI | p-value | Odds ratio | 95%CI | Standard errors | p-value | Odds ratio | 95%CI | Robust standard errors | p-value |
| Direct Causes |  |  |  |  |  |  |  |  |  |  |  |  |
| **Deaths due to AIDS/TB** | Yes | 2.92 | 1.83 - 4.66 | <0.001 | 7.54 | 3.03 -18.8 | 3.510 | <0.001 | 7.54 | 7.49-7.59 | 0.0277 | <0.001 |
|  | No | 1 |  |  | 1 |  |  |  |  |  |  |  |
| **Deaths due to unknown causes** | Yes | 1.24 | 0.45- 3.42 | 0.680 |  |  |  |  |  |  |  |  |
|  | No | 1 |  |  |  |  |  |  |  |  |  |  |
| **Deaths due to non-communicable diseases** | Yes | 1.01 | 0.13 -7.55 | 0.993 |  |  |  |  |  |  |  |  |
|  | No | 1 |  |  |  |  |  |  |  |  |  |  |
| Underlying causes at household/community and district levels |  |  |  |  |  |  |  |  |  |  |  |  |
| **Source of drinking water** | Borehole | 1.18 | 0.71-1.95 | 0.517 | 1.06 | 0.65-1.81 | 0.280 | 0.821 | 1.06 | 0.99-1.12 | 0.0325 | 0.054 |
|  | Well | 1.28 | 0.75 - 2.17 | 0.365 | 1.18 | 0.71-2.07 | 0.322 | 0.545 | 1.18 | 1.16-1.21 | 0.0127 | <0.001 |
|  | Surface(lakes and dams) | 1.71 | 1.23 - 2.36 | 0.001 | 1.70 | 1.21-2.32 | 0.278 | 0.001 | 1.70 | 1.70-1.72 | 0.0045 | <0.001 |
|  | Other | 1.31 | 0.91- 1.89 | 0.142 | 1.38 | 0.92-1.91 | 0.248 | 0.071 | 1.38 | 1.37-1.39 | 0.0048 | <0.001 |
|  | Piped water | 1 |  |  |  |  |  |  | 1 |  |  |  |
| **Mother HIV Positive** | Yes | 1.08 | 0.81 - 1.45 | 0.584 |  |  |  |  |  |  |  |  |
|  | No | 1 |  |  |  |  |  |  |  |  |  |  |
| **Father HIV Positive** | Yes | 1.24 | 0.62 - 2.49 | 0.538 |  |  |  |  |  |  |  |  |
|  | No | 1 |  |  |  |  |  |  |  |  |  |  |
| **Wealth Index** | Poor | 0.95 | 0.71- 1.29 | 0.787 |  |  |  |  |  |  |  |  |
|  | Middle | 0.90 | 0.64 - 1.26 | 0.547 |  |  |  |  |  |  |  |  |
|  | Rich | 1 |  |  |  |  |  |  |  |  |  |  |
| **Ever vaccination** | No | 0.99 | 0.81 - 1.22 | 0.968 |  |  |  |  |  |  |  |  |
|  | Yes | 1 |  |  |  |  |  |  |  |  |  |  |
| **Mother education** | None | 0.57 | 0.21-1.55 | 0.272 |  |  |  |  |  |  |  |  |
|  | Primary | 0.76 | 0.55- 1.04 | 0.084 |  |  |  |  |  |  |  |  |
|  | Secondary or more |  |  |  |  |  |  |  |  |  |  |  |
| **Birth order** | First | 0.99 | 0.82- 1.20 | 0.964 |  |  |  |  |  |  |  |  |
|  | Five or higher | 0.91 | 0.64 - 1.29 | 0.596 |  |  |  |  |  |  |  |  |
|  | Second, Third and Fourth | 1 |  |  |  |  |  |  |  |  |  |  |
| **Distance from nearest clinic in km** | >10 | 1.27 | 0.55 - 2.93 | 0.568 |  |  |  |  |  |  |  |  |
|  | <10 |  |  |  |  |  |  |  |  |  |  |  |

**Table B: KULLDORF MULTIVARIABLE LOGISTIC REGRESSION**

|  |  | **Univariate** | | | **Maximum likelihood** | | | | **Huber’s Adjustment** | | | |
| --- | --- | --- | --- | --- | --- | --- | --- | --- | --- | --- | --- | --- |
| Characteristic |  | Odds ratio | 95%CI | p-value | Odds ratio | 95%CI | Standard errors | p-value | Odds ratio | 95%CI | Robust standard errors | p-value |
| Direct Causes |  |  |  |  |  |  |  |  |  |  |  |  |
| **Deaths due to AIDS/TB** | Yes | 1.93 | 1.30- 2.87 | 0.001 | 2.26 | 0.64-7.90 | 1.4429 | 0.204 | 2.26 | 2.16 - 2.35 | 0.0487 | <0.001 |
|  | No | 1 |  |  |  |  |  |  |  |  |  |  |
| **Deaths due to unknown causes** | Yes | 1.14 | 0.09 -1.98 | 0.748 |  |  |  |  |  |  |  |  |
|  | No | 1 |  |  |  |  |  |  |  |  |  |  |
| **Deaths due to non-communicable diseases** | Yes | 1.02 | 0.67-2.30 | 0.967 |  |  |  |  |  |  |  |  |
|  | No | 1 |  |  |  |  |  |  |  |  |  |  |
| Underlying causes at household/community and district levels |  |  |  |  |  |  |  |  |  |  |  |  |
| **Source of drinking water** | Borehole | 1.04 | 0.73 - 1.49 | 0.809 | 0.77 | 0.50- 1.17 | 0.1664 | 0.224 | 0.77 | 0.76- 0.77 | 0.0010 | <0.001 |
|  | Well | 0.89 | 0.59 -1.36 | 0.596 | 0.78 | 0.49- 1.24 | 0.1828 | 0.290 | 0.78 | 0.77- 0 .79 | 0.0035 | <0.001 |
|  | Surface(lakes and dams) | 0.89 | 0.68 - 1.17 | 0.416 | 0.92 | 0.69 - 1.21 | 0.1296 | 0.533 | 0.92 | 0.91- 0.92 | 0.0001 | <0.001 |
|  | Other | 1.29 | 1.01-1.65 | 0.040 | 1.27 | 0.99- 1.63 | 0.1622 | 0.071 | 1.27 | 1.25 - 1.29 | 0.0108 | <0.001 |
|  | Piped water | 1 |  |  |  |  |  |  | 1 |  |  |  |
| **Mother HIV Positive** | Yes | 1.06 | 0.86 -1.31 | 0.584 |  |  |  |  |  |  |  |  |
|  | No | 1 |  |  |  |  |  |  |  |  |  |  |
| **Father HIV Positive** | Yes | 1.37 | 0.88- 2.13 | 0.162 |  |  |  |  |  |  |  |  |
|  | No | 1 |  |  |  |  |  |  |  |  |  |  |
| **Wealth Index** | Poor | 0.97 | 0.78 - 1.20 | 0.757 |  |  |  |  |  |  |  |  |
|  | Middle | 1.33 | 0.76 - 1.65 | 0.412 |  |  |  |  |  |  |  |  |
|  | Rich | 1 |  |  |  |  |  |  |  |  |  |  |
| **Ever vaccination** | No | 1.02 | 0.88 - 1.17 | 0.810 |  |  |  |  |  |  |  |  |
|  | Yes | 1 |  |  |  |  |  |  |  |  |  |  |
| **Mother education** | None | 0.43 | 0.19- 1.98 | 0.545 |  |  |  |  |  |  |  |  |
|  | Primary | 0.95 | 0.77 - 1.18 | 0.658 |  |  |  |  |  |  |  |  |
|  | Secondary or more | 1 |  |  |  |  |  |  |  |  |  |  |
| **Birth order** | First | 1.02 | 0.89 - 1.17 | 0.749 |  |  |  |  |  |  |  |  |
|  | Five or higher | 1.06 | 0.84 - 1.33 | 0.607 |  |  |  |  |  |  |  |  |
|  | Second, Third and Fourth | 1 |  |  |  |  |  |  |  |  |  |  |
| **Distance from nearest clinic in km** | >10 | 0.89 | 0.47- 1.72 | 0.736 |  |  |  |  |  |  |  |  |
|  | <10 |  |  |  |  |  |  |  |  |  |  |  |

**Table C: Profile of clustering vs. non-clustering cases in terms of risk factors.**

| **Risk factors** | **Clustering** | |
| --- | --- | --- |
|  | **Cluster (%)** | **Non-cluster (%)** |
| AIDS AND TB | 13.5 | 86.5 |
| Unknown causes | 6.1 | 93.9 |
| Non-communicable diseases | 5 | 95 |
| **Water source** |  |  |
| Piped water | 4.6 | 95.4 |
| Borehole | 5.4 | 94.6 |
| Well | 5.9 | 94.1 |
| Surface water bodies | 7.7 | 92.3 |
| Other | 6 | 94 |
| Mother HIV Positive | 5.5 | 94.5 |
| Father HIV Positive | 5.2 | 94.8 |
| **Wealth index** |  |  |
| Rich | 5.4 | 94.6 |
| Middle | 4.9 | 95.1 |
| Poor | 5.2 | 94.8 |
| Ever had vaccination | 4.8 | 95.2 |
| **Education** |  |  |
| Secondary or more | 5.4 | 94.6 |
| Primary | 4.1 | 95.9 |
| None | 3.1 | 96.9 |
| **Birth order** |  |  |
| Five or higher | 4.8 | 95.2 |
| Second ,Third and Fourth | 5.2 | 94.8 |
| First | 5.2 | 94.8 |
| **Distance to nearest clinic** |  |  |
| Less than 10 km | 5 | 95 |
| Greater than or equal to 10km | 6.3 | 93.7 |

**Table D : Space Time analysis Output**

1.Location IDs included :11336,11324,11304 ,11361, 11309

Coordinates / radius... :(28.428631 S, 32.172039 E) / 0.043 km

Time frame………………..: 2008/1/1 to 2014/12/31

Annual cases / 100000.: 12903.4

Observed / expected…: 13.56

Relative risk………………….13.59

Log likelihood ratio………:8.409379

P-value………………………….:0.91

Recurrence interval………:1.1 years

**Figure A: ROC curve to assess the predictive ability of the model**


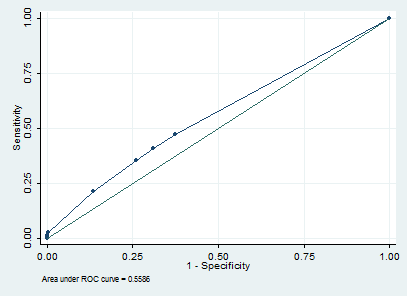

Supplement: S1 Appendix — (DOCX) [file pone.0182478.s002.docx]
